# Is it time to stock up? Understanding panic buying during the COVID-19 pandemic

**DOI:** 10.1080/00049530.2023.2180299

**Published:** 2023-03-16

**Authors:** Karina T. Rune, Jacob J. Keech

**Affiliations:** a School of Health, University of the Sunshine Coast, Sippy Downs, Australia; b School of Applied Psychology, Griffith University, Brisbane, Australia

**Keywords:** COVID-19, behavioural triggers, hoarding

## Abstract

**Background:**

Lockdowns to reduce the spread of COVID-19 have triggered sharp increases in consumer purchasing behaviour, labelled panic buying. Panic buying has detrimental consequences as it leads to product shortages and disrupts supply chains, forcing retailers to adopt quotas to manage demand. Developing an understanding of the psychological correlates of panic buying can provide targets for public messaging aimed at curbing the behaviour.

**Objective:**

The study aimed to identify the psychological, individual difference, and demographic factors associated with increased purchasing of non-perishable, cleaning, and hygiene products during COVID-19 lockdowns in Australia.

**Methods:**

The study used a cross-sectional design (*N* = 790) with online survey measures administered to community members in Australia during April and May 2020. Data were analysed using structural equation modelling.

**Results:**

Structural equation models revealed that 1) attitudes, subjective norms, and risk perceptions predicted increased purchasing of non-perishable products; 2) attitudes, risk perceptions, social anxiety sensitivity, and the non-impulsivity facet of trait self-control predicted increased purchasing of hygiene products; and 3) attitudes and risk perceptions predicted increased purchasing of cleaning products.

**Conclusion:**

Findings provide an understanding of the factors that were associated with panic buying during COVID-19 lockdowns in Australia. Future studies should investigate whether messages designed to influence risk perceptions, attitudes, and subjective norms are effective in curbing the behaviour.

## Key Points

**What is already known about this topic:**

1. Lockdowns to curb the spread of COVID-19 prompted substantial increases in consumer purchasing behaviour, labelled panic buying.
2. Prior research had identified a range of individual difference factors as being associated with panic buying, including intolerance of uncertainty and distress intolerance.
3. Identification of modifiable psychological processes, which are associated with the behaviour, is needed to inform public messaging aimed at curbing the behaviour.

**What this topic adds:**

1. The study provides information from a large national sample of Australians who regularly purchase groceries.
2. Our results suggest that potentially modifiable social cognition factors were most closely associated with increases in consumer purchasing behaviour when COVID-19 lockdowns were announced.
3. Public messaging should target attitudes, subjective norms, and risk perceptions regarding increased purchasing behaviour and future research should evaluate the effect of such messaging.

## Introduction

Since December 2019, the world has lived through “uncharted territory” (World Health Organization [WHO], 2020) with the spread of the SARS-CoV-2 novel coronavirus (COVID-19) and the resultant pandemic. The COVID-19 pandemic impacted consumer behaviour throughout the world with reports of *panic buying* and stockpiling of household commodities leading to temporary shortages (O’Connell et al., 2020; Sim et al., 2010). The increase in consumer purchasing behaviour, labelled as panic buying or hoarding in the media, refers to buying and stockpiling more of everyday household items than is necessary to sustain a household during routine life, and in anticipation of a potential future shortage (Bentall et al., 2021; Shou et al., 2013; Yoon et al., 2018). Panic buying becomes problematic when it disrupts production and supply chains (Shou et al., 2013), creating demand-side scarcity, which forces retailers into adopting quotas and price increases (Woertz, 2010).

During the first wave of the COVID-19 pandemic in Australia (April – May 2020), the media actively attempted to discourage consumers from engaging in panic buying. However, research indicates that the media may have exacerbated the situation and made people more anxious by using sensationalist headlines, showing pictures of people engaged in panic buying, long queues, and empty supermarket shelves (Arafat et al., 2020; Dubey et al., 2020; Nicola et al., 2020). This phenomenon has been referred to as the *scarcity effect* whereby people observe the behaviour of others to estimate the seriousness of a crisis (Arafat et al., 2020; Pantano et al., 2020). Additionally, a key reason for the inefficacy of media messaging for behaviour change is that these messages are often largely atheoretical and do not target the psychological processes that underpin the behaviours (Randolph & Viswanath, 2004; Webb et al., 2010). Understanding theoretically based psychological processes will support the identification of behaviour change methods that have been shown to be effective for changing human behaviour (Hagger et al., 2020).

Panic buying is an unusual and rare event that is difficult to study retrospectively. A study conducted in Singapore during the 2003 SARS outbreak reported substantial increases in psychiatric morbidities including somatic disorders, anxiety, depression, and social dysfunction (Sim et al., 2010). Participants in the study expressed concerns about a loss of control, fear of contagion, unpredictability of the situation, impact on the economy, and family health concerns. In addition, younger age and being female were associated with greater anxiety. A systematic review by Yuen et al. (2020) identified that panic buying in health crises prior to the COVID-19 pandemic was influenced by four factors: (1) perception of threat, (2) fear of the unknown, (3) coping behaviour, and (4) social psychological factors. The authors concluded that when consumers perceive the probability and consequences of contracting a disease as high, self-motivated behaviours, such as panic buying, increase to mitigate perceived risk. In 2021, Labad et al. (2021) published a systematic review on 14 recent studies that had investigated toilet paper hoarding prior to the COVID-19 pandemic. The review found that social media and social cognitive biases were potential contributors to toilet paper stockpiling. Further, psychological factors such as situational stress, fear of contagion and personality traits (e.g., conscientiousness and emotionality) were associated with panic buying behaviour. These studies suggest that panic buying is a complex behaviour, however, there is currently limited literature on the social cognition variables underpinning panic buying. Understanding these influences is important as they may support the development of targeted public campaigns, framed with personally relevant information to help alleviate the perceived need to engage in panic buying.

The COVID-19 pandemic has been a time of uncertainty for many with extended periods of increased social isolation. In contrast to disaster preparation (e.g., floods or hurricanes), where people are more aware which items to purchase, panic buying appears to be largely impulsive and driven by uncertainty. While conceptually different, hoarding and panic buying behaviours have been theorised to be driven by the same psychological mechanisms fermented in an instinctive evolutionary reaction to a perceived threat (Cameron & Shah, 2015) and motivated by a fear of being caught unprepared (Frost & Gross, 1993). In a survey of 54 countries, Keane and Neal (2021) found that bursts of panic buying typically lasted 7 to 10 days and were linked to government announcements of movement restrictions (e.g., lockdowns). Research into hoarding behaviour more broadly suggests that individual difference factors such as distress intolerance and intolerance of uncertainty may influence panic buying (Grisham et al., 2018; Norberg et al., 2015). In an attempt to reduce feelings of insecurity and uncertainty, people may gravitate towards things they can control, such as purchasing products that fulfill basic needs (Dubey et al., 2020; Prentice et al., 2020; Sim et al., 2010; Yoon et al., 2018).

Individuals have reported up to a 49% increase in anxiety concerning their safety and livelihood during the COVID-19 pandemic (Evidation, 2020). Such unease may turn individuals to coping strategies aimed at exerting control over an uncontrollable situation (Hori & Iwamoto, 2014). Taylor et al. (2020) reported that people who are extremely worried about COVID-19 are more likely to engage in panic buying. These people also tend to be high in intolerance to uncertainty. Similarly, research conducted during the very early stages of the COVID-19 pandemic in the UK (N = 2,025) and Ireland (N = 1,041) reported that psychological distress and anxiety (death anxiety and threat sensitivity) predicted over-purchasing across a wide range of products (Bentall et al., 2021). Weismuller et al. (2020) investigated correlates of COVID-19 adherent and dysfunctional (e.g., panic buying) safety behaviours in over 15,000 German participants. The study found that panic buying was a psychological reaction to a current crisis and the fear of an interruption to the supply chain. Similarly, Herjanto et al. (2021) found that situational ambiguity and evaluation-based thinking style increased perceived risk, which in turn generated panic buying. Given these findings, it is essential to further investigate factors associated with panic buying behaviour in an Australian context as this will aid in the development of campaigns aimed at decreasing such behaviour so that supply chains can manage stock levels.

Emerging research suggests that social psychological and demographic variables may also impact panic buying behaviour. For example, Bentall et al. (2021) found that household income and children in the home predicted increased panic buying behaviour. Similarly, Dinić and Bodroža (2021) reported that gender, age, and educational level (N = 545) were not related to stockpiling, whereas household size positively correlated with stockpiling. Results from a Brazilian study found that panic buying happens in every income class, but that a positive correlation exists between average income per capita and panic buying (Yoshizaki et al., 2020). Further, social influence (e.g., via the media, online, or in person) and distrust have been proposed to influence consumer behaviour in reaction to legislation and restrictions put in place by the government (Loxton et al., 2020; Zheng et al., 2021). This is proliferated by mimicking influential others and knee jerk reactions to social media posts of panic buying and empty supermarket shelves (Zheng et al., 2021).

### An integrated social cognition approach

While the research conducted to date has provided an indication of a range of factors that are associated with panic buying behaviour, using theory to identify modifiable psychological processes underpinning these behaviours can help to ascertain the optimal content of messages aimed at reducing engagement in the behaviour. Theoretical messages are widely supported as being more effective and more straightforward to evaluate than atheoretical messages for changing behaviour (Webb et al., 2010). Social cognition theories have a long tradition of being applied to changing behaviour relevant to health and in health-related contexts (Hagger et al., 2020). A prototypical social cognition model is the theory of planned behaviour (Ajzen, 1991). Within the theory, *behavioural intention* (a person’s readiness to engage in a given behaviour), is the most proximal determinant of behaviour. Behavioural intention is determined by *attitudes* towards the behaviour (positive or negative evaluations of the behaviour), *subjective norms* (perceived social pressure to engage in the behaviour), and *perceived behavioural control* (perceived ability to perform the behaviour).

While the theory of planned behaviour is a well-established and parsimonious approach to explaining health-related behaviour (Armitage & Conner, 2001; McEachan et al., 2011), it has some notable limitations. First, the theory constructs consistently explain considerably more variance in intention than behaviour. That is, those who intend to engage in a behaviour often do not follow through with their intention (Orbell & Sheeran, 1998). This is known as the intention-behaviour gap. Second, the theory only seeks to account for conscious and reasoned processes. Finally, a large portion of variance in behaviour remains unexplained by theory constructs. To overcome these limitations, researchers have sought to integrate complementary constructs from other social cognition theories. One such approach is to include an index of behavioural automaticity to account for the potential impulsive nature of behaviour. This draws from dual-process models of cognition and behaviour (Evans & Stanovich, 2013; Strack & Deutsch, 2004), which posit that behaviour is a function of two independent systems working in parallel – a *reflective* system whereby behaviour is regulated through reasoned conscious decision-making, and an *impulsive* system whereby behaviour is regulated through automatic and non-conscious decision-making.

Including behavioural automaticity in integrated social cognition models has been found to explain unique variance in a range of health-related behaviours (Brown et al., 2020; Hamilton et al., 2020; Phipps et al., 2021), including transmission prevention behaviours during the COVID-19 pandemic (Hagger, Smith, et al., 2021). Another complementary construct that has been commonly included in integrated social cognition models is the affective construct known as risk perceptions. *Risk perceptions* are beliefs regarding personal risk or susceptibility to certain outcomes or conditions if engaging or not engaging in a particular behaviour. Risk perceptions form a part of two theories that have widely been applied to understanding health-related behaviour, namely the health belief model (Rosenstock, 1974) and the health action process approach (Schwarzer, 2008).

Applying an extended theory of planned behaviour, Lehberger et al. (2021) found that attitudes, subjective norms, and fear of future unavailability were the main predictors of stockpiling of non-perishable foods in Germany. Similarly, Roșu et al. (2021) examined stockpiling during COVID-19 lockdowns in Romania. Their findings suggested that attitudes and social norms predicted both intentions to stockpile and actual stockpiling behaviours. While this research has started to map the social cognition factors associated with panic buying, no research to date has explored the predictors of panic buying in an Australian context. In addition, no research has examined and compared the predictors of panic buying across product categories. Increasing the precision of measuring the behaviour to specify specific product categories provides the opportunity to determine whether different factors are associated with changes in different types of products. To date, no research has made this distinction in the context of panic buying during the COVID-19 pandemic. This information from the Australian context attained through measurement of behaviour in relation to specific product categories can provide important insight into the optimal content for public messaging to prevent panic buying during future similar health-related events in Australia.

### The present study

**Figure 1.**
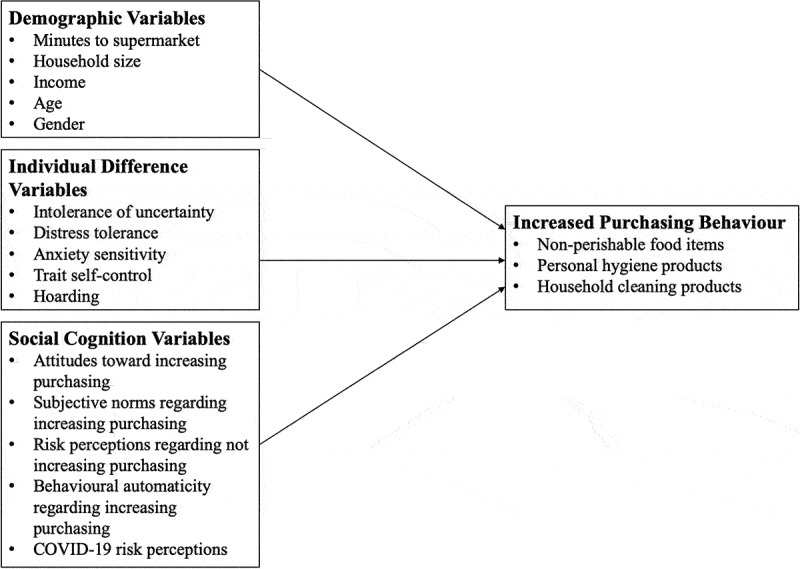
Conceptual map of the research hypotheses.

The current study aimed to identify the psychological and individual difference factors that are associated with increased purchasing of (1) non-perishable food items, (2) personal hygiene products, and (3) household cleaning products during the COVID-19 pandemic of 2020. First, based on prior research into similar behaviours (e.g., Bentall et al., 2021; Grisham et al., 2018), it was hypothesised that individual difference factors including intolerance of uncertainty, distress tolerance, anxiety sensitivity, trait self-control, hoarding rating, and COVID-19 risk perceptions, will be associated with increases in each purchasing behaviour. Second, drawing upon an integrated social cognition theoretical approach, it was hypothesised that attitudes, subjective norms, risk perceptions, and behavioural automaticity will be associated with increases in purchasing behaviour. Third, it was hypothesised that demographic factors including minutes to the supermarket, household size, household income, age, and gender, will predict each purchasing behaviour. Refer to Figure 1 for a conceptual map of the study hypotheses.

## Method

### Participants

A total of 821 participants were recruited across the general Australian population. Participants were eligible to participate in the study if they regularly purchased food or other household items from the supermarket and were over 18 years of age. A total of 15 participants were excluded due to not meeting eligibility criteria (2 based on age and 13 based on not regularly purchasing). Due to inattentive responding, 31 participants were also systematically excluded, leaving a final sample of 790 participants. The age range of participants was 18 to 86 years (*M*_age_ = 48.89 years *SD* = 13.23), with 613 participants identifying as female, 173 as male and 4 as a different gender. Participation was voluntary and responses were anonymous. No incentives were offered to participate in the study. Detailed demographic information can be found in the Table 1.

**Table 1.** Demographic information relating to frequency and percentage of participants in the study (N = 790).

	Frequency	Percentage		Frequency	Percentage
**Ethnicity**			**Education level**		
Australian	562	71.1	Year 10	67	8.5
Aboriginal Australian	2	.3	Year 12	82	10.4
European/Caucasian	71	9.0	TAFE, Certificate/Diploma, trade	255	32.3
Asian	12	1.5	or VET Qualification		
Other	13	1.6	Bachelor’s degree	196	24.8
**Country of birth**			Post graduate degree	187	23.7
Australia	559	70.8	**Currently studying a degree at university**		
Africa	14	1.8	Yes	98	12.4
Asia	19	2.4	No	690	87.3
Canada	7	.9	**Employment**		
Europe	28	3.5	Full-time	309	39.1
Middle East	3	.4	Part-time	111	14.1
New Zealand	24	3.0	Unemployed/home duties	58	7.3
United Kingdom	60	7.6	Unemployed looking for work	20	2.5
United States of	28	.5	Unemployed not looking for work	11	1.4
America			Retired	129	16.3
**Marital status**			Full-time student	23	2.9
Never married	113	14.3	Studying and working	29	3.7
In a relationship	53	6.7	Disabled	26	3.3
Married	398	50.4	Currently not working due to COVID-19	52	6.6
De-facto	81	10.3	**Weekly (annual) household income**		
Separated/divorced	115	14.6	Nil income	14	1.8
Widowed	28	3.5	$1–$199 ($1-$10.399)	11	1.4
**Children**			$200–$299 ($10,400-$15,599)	14	1.8
Yes	538	68.1	$300–$399 ($15,600-$20,799)	41	5.2
No	246	31.1	$400–$599 ($20,800–31,199)	53	6.7
**Children living at home**			$600–$799 ($31,200-$41,599)	67	8.5
0	328	41.5	$800–$999 ($41,600-$51,999)	49	6.2
1	123	15.6	$1000–$1249 ($52,000-$64,999)	70	8.9
2	145	18.4	$1250–$1499 ($65,000-$77,999)	69	8.7
3	67	8.5	$1,500–$1,999 ($78,000-$103,999)	131	16.6
4	18	2.3	> $2000 (>$104,000)	256	32.4
5+	9	1.0			
**Number of people in**			**Minutes to shop**		
**household**			1–10 minutes	620	86.1
1	132	16.7	1–20 minutes	76	9.6
2	293	37.1	21–30 minutes	25	3.2
3	118	14.9	31–40 minutes	2	.3
4	130	16.5	41–50 minutes	3	.4
5	69	8.7	51–60 minutes	1	.1
6+	35	4.5	>60 minutes	3	.4

Missing responses are not recorded. Information is based on the final sample of 790 participants after careless respondents were removed.

### Design and procedure

The University Human Research Ethics Committee approved the study (protocol: A201375). The study used a cross-sectional survey design. Participants were recruited across Australia through methods which included the research team speaking about the study on television and radio news broadcasts. The researchers were also interviewed about the study and quoted in online and print newspaper articles. All media stories about the study included a link to participate in the survey. Participants were also recruited online using social media and snowball sampling. Data were collected during the height of the COVID-19 pandemic in Australia (April and May 2020). Participants were informed the study was investigating attitudes and beliefs towards stocking up on groceries during the COVID-19 pandemic. The survey package included two screening questions (i.e., “do you regularly purchase food or other household items from the supermarket” and “age>18 years”). Ineligible participants were exited from the survey. Eligible participants were asked to complete the online survey hosted by Qualtrics survey software. The survey remained open for 5 weeks and sample size was determined based on this recruitment window.

### Measures

Item wording for all measures is provided in Supplementary Appendix A. Where item wording could not be reproduced due to copyright, the supplementary material contains information regarding where the items can be accessed. Revelle’s ω was calculated using the *userfriendlyscience* (Peters, 2017) package in *R* (R Core Team, 2019) as a measure of reliability for each scale (McNeish, 2018; Peters, 2017). Reliability for two-item scales was calculated using Spearman rank order correlations. All scales exhibited satisfactory reliability. See Supplementary Appendix B for reliability coefficients.

#### Demographic variables

The following demographic variables were assessed: gender; marital status; number of children; number of children living at home; number of people living in household; education levels; currently studying an undergraduate degree; employment status; hours in paid employment; field of study and/or work; and weekly (annual) income.

#### Purchasing behaviour

Participants’ increases in purchasing behaviour was assessed using a single item measure for each of the following three behaviours (referred to as a category in the sample measurement wording below). The first behaviour was defined as increased purchasing of non-perishable food items (e.g., pastas, rices, drinks, canned, flour, sugar, frozen vegetables, pet food etc.). The second behaviour was defined as increased purchasing of hygiene products (e.g., hand sanitiser, bleach, wipes, disinfectant, washing powder etc.). The third behaviour was defined as increased purchasing of cleaning products (e.g., toilet paper, tissues, nappies, nappy wipes, etc.). Participants were advised that the questions would ask them to indicate the extent to which they have bought more products than they would use based on their usual frequency of shopping, since the COVID-19 pandemic began this year, regarding each category. Responses were provided on 7-point scales (1 = “*I have bought only the amount of <category> products that I usually buy*”; scales 2–7 started with “*I have increased my purchasing to buy enough <category> products for …* ” 2 = “*an extra few days*”, 3 = “*an extra week*”, 4 = “*an extra two weeks*”, 5 = “*an extra three weeks*”, 6 = “*an extra month*”, 7 = “*more than an extra month*”. Given that an established scale did not exist for this purpose, we applied best practice principles for self-report measurement of behaviour such as ensuring behavioural definitions and rating scales are clearly worded in a manner that is specific regarding target, action, context, and time (Ajzen, 2006a). Single item measures of behaviour have been found to be valid in other relevant contexts such as retrospective recall and self-reporting of physical activity (Hamilton et al., 2012).

#### Social cognition constructs

Measures of social cognition constructs were adapted to the current behavioural context based on established guidelines (Ajzen, 1991, 2006b; Gardner et al., 2012).

##### Attitudes

Attitudes towards each behaviour were assessed using three items preceded by a common stem: “If I were to buy more non-perishable products than I would use based on my usual frequency of shopping, it would be”:. Responses were provided on semantic differential scales (e.g., 1 = *bad* and 7 = *good*). The scale was administered for each behaviour.

##### Subjective norms

Subjective norms for each behaviour were measured using five items (e.g., “Most people who are important to me would approve of me buying more non-perishable products”.). The scale was administered for each behaviour. Responses were provided on 7-point scales (1 = *strongly disagree* and 7 = *strongly agree*).

##### Risk perceptions

Risk perceptions for each behaviour were measured using two items (e.g., “It would be risky for me not to buy more non-perishable products”). The scale was administered for each behaviour. Responses were provided on 7-point scales (1 = *strongly disagree* and 7 = *strongly agree*).

##### Behavioural automaticity

Behavioural automaticity was measured using the four-item behavioural automaticity subscale (Gardner et al., 2012) of the Self-Report Habit Index (Verplanken & Orbell, 2003). The measure asks respondents to reflect on their agreement with statements regarding their enactment of the behaviour automatically and without the need for conscious thought. The scale was administered for each behaviour. Responses were provided on 7-point scales (1 = *strongly disagree* and 7 = *strongly agree*).

#### Individual difference constructs

##### Anxiety sensitivity

The Anxiety Sensitivity Index-3 (ASI-3; Taylor et al., 2007) is an 18-item questionnaire measuring fear of arousal-related sensations across three empirically established subscales with 6 items each. The subscales relate to physical (e.g., “when my stomach is upset, I worry that I might be seriously ill”), cognitive (e.g., “when my thoughts seem to speed up, I worry that I might be going crazy”, and social concerns (e.g., “it is important for me not to appear nervous”). The scale utilises a 5-point Likert scales from 0 (*very little*) to 4 (*very much*). Total scores are summed and range from 0 to 72 with higher scores indicating greater arousal-related sensation. The scale has demonstrated excellent psychometric properties, with coefficient alpha across the subscales ranging from .73 to .91 in cross-cultural norm groups (Taylor et al., 2007).

##### Distress tolerance

The Distress Tolerance Scale (DTS; Simons & Gaher, 2005) is a 15-item scale examining ability to tolerate psychological distress rated on a 5-point Likert scale with response options ranging from 1 (*strongly agree*) to 5 (*strongly disagree*). The scale has four subscales: tolerance (3 items e.g., “I can’t handle feeling distressed or upset”), appraisal (6 items e.g., “my feelings of distress or being upset scare me”), absorption (3 items e.g., “my feelings of distress are so intense that they completely take over”), and regulation (3 items e.g., “I’ll do anything to avoid feeling distressed or upset”). Lower scores indicate a tendency to experience psychological distress as intolerable. Scores on the DTS have been shown to be negatively correlated with measures of negative affectivity and lability and positively correlated with measures of positive affectivity (Simons & Gaher, 2005). The scale has demonstrated test-retest stability over six months (Simons & Gaher, 2005).

##### Intolerance of uncertainty

The Intolerance of Uncertainty Scale, Short From (IUS-12; (Carleton et al., 2007) is a short-form of the original 27-item Intolerance of Uncertainty Scale (Buhr & Dugas, 2002; Freeston et al., 1994), rated on a 5-point Likert scale from 1 (*not at all characteristic of me*) to 5 (*entirely characteristic of me*), which measures reactions to uncertainty, ambiguous situations, and the future. The IUS-12 has two subscales. The 7-item prospective anxiety subscale measures anxiety in anticipation of uncertainty (e.g., “I can’t stand being taken by surprise”), whereas the 5-item inhibitory anxiety subscale measures inhibition of action or experiences (e.g., “I must get away from all uncertain situations”). Higher scores are indicative of greater intolerance of uncertainty. Both subscales have been found to have similarly high internal consistency, α = .85 (Carleton et al., 2007). The IUS-12 has also been found to be highly correlated (*r* = .96) with the full version in two studies with both student (Carleton et al., 2007) and clinical samples (McEvoy & Mahoney, 2011).

##### Self-control

The Brief Self-Control Scale (BSCS; Tangney et al., 2004) was developed to assess dispositional self-control across 13 items rated on a 5-point Likert scale from 1 (*not at all*) to 5 (*very much*). Due to underlying concerns with the validity of its standard unidimensional structure, an updated approach to scoring the Brief Self-Control Scale was utilised (Maloney et al., 2012). Maloney's et al. (2012) two-factor model includes 4-items measuring restraint (i.e., items 1, 2, 7, 8 from the original BSCS) and 4 items measuring impulsivity (i.e., items 5, 9, 12, 13 from the original BSCS). Examples of subscale items are “I am good at resisting temptation” and “I have trouble concentrating”, respectively. Scores are averaged, with higher scores indicative of greater self-control. Hagger, Zhang, et al. (2021) found that the two-factor model had the best psychometric properties across four international samples.

##### Hoarding

The Hoarding Rating Scale (HRS; Tolin et al., 2010) is a brief self-administered scale that assesses the features of compulsive hoarding, which includes five questions covering clutter, difficulty discarding, acquisition, distress, and impairment. Each question is measured on a 9-point Likert scale ranging from 0 (*none*) to 8 (*extreme*). When scores are averaged, a score of 4 represents moderate symptoms. The HRS correlates strongly with the interview version of the scale and both scales have excellent psychometric properties (internal consistency, test-retest reliability, and interrater reliability; Tolin et al., 2010).

#### Perceived risks surrounding COVID-19

##### COVID-19 risk perceptions

A measure of COVID-19 risk perceptions was developed based on the core components of risk perceptions identified by Brewer et al. (2007): risk likelihood, susceptibility, and severity (e.g., “If I got COVID-19, there is a good chance that I would have trouble”). Responses were provided on 7-point scales (1 = *strongly disagree* and 7 = *strongly agree*).

#### Data quality questions

Two questions were used to detect inattentive responding (e.g., please select option two to ensure you are paying attention; Maniaci & Rogge, 2014; Schroder et al., 2016). The 31 participants who did not answer the two questions correctly were excluded prior to data analysis.

### Data analysis

The three hypothesised models were evaluated using latent variable structural equation modelling in the *lavaan* package (Rosseel, 2012) in R (R Core Team, 2019). Due to the presence of multivariate skewness (determined based on ratio of skewness to skewness *SE*>3.29), the MLR estimator was used for all analyses. The MLR estimator is a maximum likelihood estimator that implements robust standard errors based on the Huber-White method (Rosseel, 2012). There were no missing data on behavioural outcome variables, however, 5.3% of participants had a small amount of missing data on the social cognition predictor variables. Missing data were estimated using the full information maximum likelihood (FIML) procedure consistent with best practice (Enders & Bandalos, 2001; Enders, 2021). Goodness of fit of the hypothesised models was evaluated using multiple criteria which compare the proposed model to the baseline model. This included Tucker-Lewis index (TLI) and comparative fit index (CFI), which should have values close to or exceeding .95; standardised root mean squared residual (SRMR), which should have a value less than .08; and root mean squared error of approximation (RMSEA), which should have a value less than .06 (Hu & Bentler, 1999). In addition, Bentler (1990) suggested that TLI and CFI statistics between .90 and .95 are indicative of acceptable model fit. An initial model was estimated for each of the three behaviours with all predictors entered. Because the default assumption of uncorrelated residuals may be a source of misfit for similarly worded items (Brown, 2015), a subsequent final model for each behaviour was estimated where residuals were allowed to covary for similarly worded and conceptually similar items that were identified as a major source of misfit by modification indices. For subjective norms, residuals for items 2 and 3, and items 4 and 5, were allowed to covary. Items 2 and 3 were components of the scale assessing injunctive norms, and used similar wording, i.e., “Those people who are important to me would want me to/think that I should buy more … ”. Items 4 and 5 were components of the scale assessing descriptive norms, and used similar wording, i.e., “people who are similar to me/like me would buy more … ”. For behavioural automaticity, residuals for items 3 and 4 were allowed to covary. Items 3 and 4 were conceptually similar in that they assessed the extent to which a behaviour is performed without thinking or having to consciously remember. For COVID-19 risk perceptions, residuals for items 1 and 2 were allowed to covary. Items 1 and 2 were conceptually similar in that they were the items within the scale assessing perceived susceptibility (as opposed to perceived severity which was assessed by the other two items). For anxiety sensitivity (cognitive), residuals were allowed to covary for items 2 and 5. Items 2 and 5 were conceptually similar in that they both assess fear and worry around inability to keep one’s mind on a task. For hoarding ratings, residuals for items 4 and 5 were allowed to covary. Items 4 and 5 were conceptually similar in that they both assess the impact of hoarding on the individual. Residual covariances were applied consistently across the models for each of the three behaviours. The data file, analysis scripts, and analysis output are available on the Open Science Framework: https://osf.io/cznj6/

## Results

Standardised path coefficients, standard errors, and 95% confidence intervals for each of the final models are presented in Table 2. Means, standard deviations, and bivariate correlations between study variables are presented in Supplementary Appendix D and E. Factor loadings for each model are presented in Supplementary Appendix F. All factor loadings were satisfactory and statistically significant. The final models including factor loadings, covariances, and path coefficients are graphically depicted in Supplementary Appendix G.

**Table 2.** Summary of standardised path coefficients, standard errors, and 95% confidence intervals for the final models for each behaviour.

	Model 1 - Non-Perishable Foods				Model 2 - Hygiene Items				Model 3 - Cleaning Items			
Effect	β	*p*	SE	95% CI	β	*p*	SE	95% CI	β	*p*	SE	95% CI
Attitude+	.30*	<.001	.05	.19, .38	.29*	<.001	.04	.19, .35	.24*	<.001	.05	.13, .33
Subjective Norm+	.12*	.024	.05	.02, .22	.07	.166	.05	−.03, .17	.03	.639	.05	−.08, .13
Risk Perception+	.32*	<.001	.05	.21, .41	.36*	<.001	.05	.25, .46	.39*	<.001	.05	.29, .49
Behavioural Automaticity+	.03	.509	.04	−.05, .11	.02	.683	.04	−.07, .10	.02	.714	.05	−.07, .11
COVID Risk Perception	.07*	.044	.07	.00, .29	.02	.536	.08	−.11, .21	.04	.275	.08	−.07, .25
Intolerance of Uncertainty – P	.04	.565	.14	−.20, .36	−.02	.842	.17	−.37, .30	.04	.636	.17	−.25, .41
Intolerance of Uncertainty – I	.07	.493	.19	−.24, .51	.22	.052	.22	−.00, .85	.05	.659	.21	−.32, .50
Distress Tolerance	.01	.829	.09	−.16, .20	.02	.644	.10	−.14, .23	−.05	.373	.10	−.28, .10
Anxiety Sensitivity – Physical	−.05	.360	.11	−.32, .12	.05	.301	.11	−.11, .34	.04	.427	.12	−.13, .32
Anxiety Sensitivity – Cognitive	.05	.319	.13	−.12, .38	.03	.573	.146	−.21, .37	−.04	.485	.15	−.40, .19
Anxiety Sensitivity – Social	−.06	.324	.15	−.45, .15	−.14*	.012	.16	−.73, −.09	−.02	.798	.18	−.40, .31
Self-control – Retraint	.01	.835	.13	−.22, .27	−.02	.681	.131	−.31, .20	.05	.382	.13	−.14, .38
Self-control – Nonimpulsivity	.02	.722	.15	−.23, .34	.12*	.038	.05	.02, .61	.07	.224	.15	−.11, .49
Hoarding Rating	−.09*	.041	.05	−.20, −.00	−.04	.369	.05	−.16, .06	−.03	.476	.05	−.13, .06
Minutes to Supermarket	.05*	<.001	.00	.00, .00	−.03*	<.001	.00	−.00, −.00	−.03*	.002	.00	−.00, −.00
Household Size	−.05	.068	.03	−.11, .00	−.03	.327	.04	−.10, .04	−.02	.582	.04	−.09, −.02
Income	.03	.321	.02	−.11, .00	.01	.630	.02	−.03, .05	−.01	.687	.02	−.05, .03
Age	.05	.083	.00	−.00, .01	.07*	.020	.00	.00, .02	.06	.054	.00	−.00, .02
Gender	.04	.215	.04	−.03, .12	.03	.364	.11	−.12, .33	.00	.990	.12	−.23, .23

+ = referenced to each behaviour. Intolerance of Uncertainty – P = IUS-12 Prospective Anxiety Subscale; Intolerance of Uncertainty – I = IUS-12 Inhibitory Anxiety Subscale; * = statistically significant based on p < .05.

### Model 1 – predictors of increased purchasing of non-perishable food items

Initial analysis of the hypothesised structure for the model with increases in purchasing of non-perishable food items as the outcome variable yielded poor model fit, χ^2^ (2365) = 5493.98, *p* < .001, CFI =.89, TLI =.88, SRMR =.05, RMSEA =.04 (90% CI [.04, .04]. Following the specification of residual covariances as described above, the final model exhibited a good fit to the data, χ^2^ (2359) = 4508.77, *p* < .001, CFI =.93, TLI =.92, SRMR =.05, RMSEA =.03 (90% CI [.03, .04]. Specifically, CFI and TLI values were close to .95, the SRMR value was below .08, and the RMSEA value was below .06, which together can be considered an indication of acceptable model fit based on Hu and Bentler’s (1999) guidelines. The modifications resulted in a significant improvement to the model fit, χ^2^ (6) = 985.21, *p* < .001; however, inferences based on the statistical significance of path estimates remained largely unchanged. The exception was that subjective norms only significantly predicted behaviour in the final model; however, and the effect size for subjective norms was consistently small across both models. The predictors in the final model accounted for 49.8% of the variance in increased purchasing of non-perishable food items. Attitudes and subjective norms regarding increased purchasing of non-perishable food items, and risk perceptions regarding not increasing purchasing of non-perishable food items, significantly predicted increased purchasing of non-perishable food items products. Risk perceptions regarding COVID-19 and the number of minutes it takes to get to the supermarket also significantly predicted increased purchasing of non-perishable food items. Finally, hoarding rating scores significantly negatively predicted increased purchasing of non-perishable food items. It should be noted that effect sizes were small for minutes to the supermarket and hoarding rating. No other variables significantly predicted increased purchasing of non-perishable food items.

### Model 2 – predictors of increased purchasing of hygiene products

Initial analysis of the hypothesised structure for the model with increases in purchasing of hygiene products as the outcome variable yielded poor model fit, χ^2^ (2365) = 5776.77, *p* < .001, CFI =.89, TLI =.88, SRMR =.05, RMSEA =.04 (90% CI [.04, .04]. Following the specification of residual covariances as described above, the final model exhibited a good fit to the data, χ^2^ (2359) = 4511.35, *p* < .001, CFI =.93, TLI =.93, SRMR =.05, RMSEA =.03 (90% CI [.03, .04]. Specifically, CFI and TLI values were close to .95, the SRMR value was below .08, and the RMSEA value was below .06, which together can be considered an indication of acceptable model fit based on Hu and Bentler’s (1999) guidelines. The modifications resulted in a significant improvement to the model fit, χ^2^ (6) = 1265.42, *p* < .001; however, inferences based on the statistical significance of path estimates remained unchanged. The predictors in the final model accounted for 49.8% of the variance in increased purchasing of hygiene products. Attitudes towards increased purchasing of hygiene products, risk perceptions regarding not increasing purchasing of hygiene products, and the non-impulsivity facet of trait self-control, significantly predicted increased purchasing of hygiene products. Social anxiety sensitivity also negatively predicted increased purchasing of hygiene products. Age also significantly positively predicted, and the number of minutes it takes to get to the supermarket significantly negatively predicted, increased purchasing of hygiene products; however, the effect sizes were small. No other variables significantly predicted increased purchasing of hygiene products.

### Model 3 – predictors of increased purchasing of cleaning products

Initial analysis of the hypothesised structure for the model with increases in purchasing of cleaning products as the outcome variable yielded poor model fit, χ^2^ (2365) = 5450.61, *p* < .001, CFI =.90, TLI =.89, SRMR =.05, RMSEA =.04 (90% CI [.04, .04]. Following the specification of residual covariances as described above, the final model exhibited a good fit to the data, χ^2^ (2359) = 4513.61, *p* < .001, CFI =.93, TLI =.92, SRMR =.05, RMSEA =.03 (90% CI [.03, .04]. Specifically, CFI and TLI values were close to .95, the SRMR value was below .08, and the RMSEA value was below .06, which together can be considered an indication of acceptable model fit based on Hu and Bentler’s (1999) guidelines. The modifications resulted in a significant improvement to the model fit, χ^2^ (6) = 937.00, *p* < .001; however, inferences based on the statistical significance of path estimates remained unchanged. The predictors in the final model accounted for 41.5% of the variance in increased purchasing of cleaning products. Attitudes towards increased purchasing of cleaning products, and risk perceptions regarding not increasing purchasing of cleaning products significantly predicted increased purchasing of cleaning products. The number of minutes it takes to get to the supermarket also significantly negatively predicted increased purchasing of cleaning products; however, the effect size was small. No other variables significantly predicted increased purchasing of cleaning products.

## Discussion

The current study identified the psychological and individual difference factors associated with increased purchasing behaviours during COVID-19 lockdowns in Australia. The study was informed by social cognition theories including the theory of planned behaviour and dual-process models, which posit that constructs such as attitudes, subjective norms, behavioural automaticity, and risk perceptions are key determinants of behaviour. Campaigns targeting such constructs are likely to be more effective for changing behaviours than atheoretical campaigns (McEachan et al., 2011). In addition, individual difference factors suggested as determinants of panic buying (Bentall et al., 2021; Labad et al., 2021; Norberg et al., 2015; Yuen et al., 2020), and demographic factors were examined as predictors of panic buying.

Consistent with our hypotheses, attitudes, and risk perceptions were supported as predictors of increased purchasing of non-perishable food items, hygiene products, and cleaning products. Subjective norms also predicted increased purchasing of non-perishable food items, although to a lesser extent. The path coefficients for the social cognition constructs, and in particular attitudes towards increasing purchasing and risk perceptions regarding not increasing purchasing, were the strongest of all predictors in each model. This is consistent with past research (e.g., Lehberger et al., 2021; Roșu et al., 2021), and suggests that social cognition constructs may play an important role in increasing purchasing behaviour during national crises. Additionally, risk perception regarding COVID-19, the number of minutes it takes to get to the supermarket, and hoarding rating were shown to predict purchasing of non-perishable food items; anxiety sensitivity, the non-impulsivity of trait self-control, age, and minutes to supermarket predicted purchasing of hygiene products; and minutes to supermarket predicted purchasing of cleaning products, albeit with considerably smaller path coefficients compared to the social cognition constructs. Together, these findings suggest that social cognition constructs may have played a more prominent role in influencing panic buying during COVID-19 lockdowns than psychological and individual difference factors. This is an important finding, given that social cognition factors are potentially modifiable through public messaging and interventions.

While the majority of the social cognition constructs, and a small number of individual difference factors and demographic factors, were supported as predictors of increased purchasing behaviours, several variables were not found to predict purchasing behaviour. Specifically, demographic variables (i.e., household size, income, age, and gender) did not significantly predict any type of purchasing behaviour in our study. This contrasts with findings by Bentall et al. (2021), who observed associations between income and panic buying in the UK and Ireland. Our findings also contrast with Dinić and Bodroža (2021), who observed associations between household size and panic buying in Serbia. This suggests that income and household size may have been less important in predicting panic buying in Australia than in other contexts, and that the behaviour was occurring across the income spectrum and across household sizes. Therefore, public messages and campaigns aimed at decreasing panic buying during national crisis events would benefit from targeting the population at large, rather than focussing on specific sub-groups. Additionally, several individual difference factors (e.g., hoarding, self-control, anxiety sensitivity, distress intolerance, intolerance of uncertainty) suggested by previous research to increase hoarding (Norberg et al., 2015) and panic buying (Bentall et al., 2021; Taylor et al., 2020) and the social cognition constructs of behavioural automaticity and COVID-19 risk perception, were not found to be significant predictors of increased purchasing behaviour, or if significant, displayed relatively small path coefficients. These findings indicate that individual difference factors proposed by current research, and variables linked to hoarding, may not be applicable to increased purchasing behaviour during COVID-19 lockdowns. Similar to recent findings by Taylor (2021), our results suggest that when aiming to decrease panic buying at the time of announcing lockdowns, focusing public messaging on variables such as self-control (e.g., “don’t panic buy” and “have some self-control”), distress, and anxiety (e.g., “calm down”) are not likely to be effective as they do not target the theoretical constructs associated with the behaviour. Finally, despite having bivariate associations with the behaviours, the finding that behavioural automaticity and COVID-19 risk perceptions did not predict increased purchasing behaviour suggests that automatic and impulsive purchasing may play a less prominent role in explaining increased purchasing behaviour surrounding lockdown announcements compared to other constructs in the model. The results of the study have yielded a range of theoretical and practical implications.

### Theoretical and practical implications

The current study advances theory in two important ways. First, the study highlights that social cognition constructs were the most important correlates of the behaviour in the context of panic buying. This is a valuable finding, as identification of social cognition constructs can directly inform the development of theory-based messages and interventions for changing behaviour (Hagger et al., 2020). Second, theory is also advanced by our integration of social cognition constructs for explaining consumer behaviour during a novel public health crisis. This specifically includes reasoned processes from the theory of planned behaviour such as attitudes and subjective norms, non-conscious processes such as behavioural automaticity, and affective processes such as risk perceptions. Such a theoretical approach can inform the prediction of behaviour, and the development of targeted messaging in the event of future COVID-19 lockdowns, or future novel public health crises.

The current study also has several important implications for practice. In particular, the results have identified three modifiable psychological processes that can be targeted in public messaging and campaigns aiming to reduce engagement in panic buying behaviour. This could be done by mapping behaviour change methods to social cognition constructs (Kok et al., 2016). First, in relation to attitudes towards panic buying, an example of a behaviour change method is the use of messaging that prompts individuals to shift their perspective (Kok et al., 2016). This could include the use of public messaging and campaigns that prompt people to imagine themselves in the shoes of frontline line works or vulnerable persons unable to buy products that have been sold out due to panic buying. Second, regarding targeting subjective norms in relation to panic buying, an example of a behaviour change method is providing information about others’ approval (Kok et al., 2016). This could include the use of public messaging that suggests that family, friends, and peers do not approve of the behaviour, and would prefer that everyone plays their part in not contributing to shortages by only buying what they need. Third, an example of a behaviour change technique targeting risk perceptions in relation to panic buying, is providing scenario-based risk information (Kok et al., 2016). This could include the use of public messaging indicating that supply chains which support grocery stores across the country are strong and that there is no risk of running out of stock if people continue to only buy what they need. To date there have been no published studies on the use of behaviour change methods in the context of panic buying. However, given the risk of future health-related crises, moving from formative research to experimental intervention research is an important future direction.

### Strengths, limitations, and future directions

The current study has several strengths that enhance our understanding of the individual difference and social cognition constructs underlying increased purchasing behaviours during COVID-19 lockdowns. First, the study applied an integrated theoretical approach grounded in the theory of planned behaviour and dual-process models to explain increased purchasing behaviour. Second, the study recruited participants from a large national sample, thus enhancing generalisability of the findings. Third, the use of structural equation modelling to analyse the data allowed for specification of latent variables and the modelling of measurement error.

The findings of the current study must also be considered in the light of some limitations. First, while the study used a large national sample, the study did not use a stratified sample and therefore cannot be assumed to be a true representation of the Australian population at large. Future research should also consider examining panic buying across countries to determine whether cultural factors are associated with the behaviour. Second, the study looked at increased purchasing of non-perishable, hygiene, and cleaning products; however, these products are not exhaustive examples of products that have been purchased during COVID-19 lockdowns. In addition, while we selected a range of individual difference factors to examine, future research could consider examining the association between further constructs such as personality factors and panic buying behaviour. Further, the study looked at only a specific time-point and did not account purchasing behaviours during prolonged lockdowns as have been seen in the Australia states of Victoria and New South Wales during 2021 and many European countries during 2020. Future research is needed to replicate the findings in response to living with COVID-19 in the future and possible short local lockdowns as more states and countries meet minimum target vaccination rates. Finally, relying on a cross-sectional retrospective design does not allow for causal inferences to be made. Future research should use experimental designs to evaluate the efficacy of a targeted intervention message aimed at changing underlying social cognition constructs to determine a causal effect, which could support public messaging campaigns aimed at decreasing the behaviour and could inform media reporting guidelines.

## Conclusion

The current study aimed to identify the individual difference factors and social cognition constructs underlying increased purchasing behaviour during COVID-19 lockdowns in Australia. The study provides insight into the processes associated with panic buying behaviours. Specifically, the results suggest that modifiable social cognition constructs including attitudes, subjective norms, and risk perception were closely associated with increased panic buying during COVID-19 lockdowns. It is recommended that these social cognition constructs be considered as targets when seeking to decrease future panic buying during health-related crises and other national crises. Future research should seek to replicate the findings during panic buying events and investigate if interventions targeting the constructs identified in this study result in causal effects on intentions to panic buy and panic buying behaviour. Such findings will enhance our understanding of panic buying during pandemics and may inform future public messaging campaigns.

## Supplementary Material

STROBE checklist cross sectional panic buying

Appendix A-F

Appendix G

## Data Availability

The data, analysis code, analysis output, and study materials are available at the Open Science Framework: https://osf.io/cznj6/.

## Open scholarship

This article has earned the Center for Open Science badges for Open Data and Open Materials through Open Practices Disclosure. The data and materials are openly accessible at https://osf.io/cznj6
