# Supplementary material for: Is it time to stock up? Understanding panic buying during the COVID-19 pandemic
Source: Aust J Psychol. 2023 Mar 16;75(1):2180299. doi: 10.1080/00049530.2023.2180299 (PMC12175686; doi:10.1080/00049530.2023.2180299)
Supplement: Appendix A-F [file RAUP_A_2180299_SM9234.docx]

Supplementary Appendix A

*Items and Reponses Scales for Study Measures*

| Variable | Item(s)/measure | Response Scale |
| --- | --- | --- |
| Purchasing Behaviour | The following questions will ask you about the extent to which you have bought more products than you would use based on your usual frequency of shopping, since the COVID-19 pandemic began this year.  We are interested in the following categories of products:  1. Non-perishable foods (e.g., pastas, rices, drinks, canned, flour, sugar, frozen vegetables, pet food etc.)  2. Cleaning products (e.g., hand sanitiser, bleach, wipes, disinfectant, washing powder etc.)  3. Hygiene products (e.g., toilet paper, tissues, nappies, nappy wipes, etc.) |  |
| Non-Perishable Products | Please select the response that best characterises your buying behaviour in relation to non-perishable foods, since the COVID-19 pandemic began: | 1 = “I have bought only the amount of non-perishable products that I usually buy”  2 = “I have increased my purchasing to buy enough non-perishable products for an extra few days”  3 = “I have increased my purchasing to buy enough non-perishable products for an extra week”  4 = “I have increased my purchasing to buy enough non-perishable products for an extra two weeks”  5 = “I have increased my purchasing to buy enough non-perishable products for an extra three weeks”  6 = “I have increased my purchasing to buy enough non-perishable products for an extra month”  7 = “I have increased my purchasing to buy enough non-perishable products for more than an extra month” |
| Hygiene Products | Please select the response that best characterises your buying behaviour in relation to hygiene products, since the COVID-19 pandemic began: | 1 = “I have bought only the amount of hygiene products that I usually buy”  2 = “I have increased my purchasing to buy enough hygiene products for an extra few days”  3 = “I have increased my purchasing to buy enough hygiene products for an extra week”  4 = “I have increased my purchasing to buy enough hygiene products for an extra two weeks”  5 = “I have increased my purchasing to buy enough hygiene products for an extra three weeks”  6 = “I have increased my purchasing to buy enough hygiene products for an extra month”  7 = “I have increased my purchasing to buy enough hygiene products for more than an extra month” |
| Cleaning Products | Please select the response that best characterises your buying behaviour in relation to cleaning products, since the COVID-19 pandemic began: | 1 = “I have bought only the amount of cleaning products that I usually buy”  2 = “I have increased my purchasing to buy enough cleaning products for an extra few days”  3 = “I have increased my purchasing to buy enough cleaning products for an extra week”  4 = “I have increased my purchasing to buy enough cleaning products for an extra two weeks”  5 = “I have increased my purchasing to buy enough cleaning products for an extra three weeks”  6 = “I have increased my purchasing to buy enough cleaning products for an extra month”  7 = “I have increased my purchasing to buy enough cleaning products for more than an extra month” |
| Social-Cognitive Variables | The following questions will ask you about buying more products than you would use based on your usual frequency of shopping, since the COVID-19 pandemic began this year.  We are interested in the following categories of products:  1. Non-perishable foods (e.g., pastas, rices, drinks, canned, flour, sugar, frozen vegetables, pet food etc.)  2. Cleaning products (e.g., hand sanitiser, bleach, wipes, disinfectant, washing powder etc.)  3. Hygiene products (e.g., toilet paper, tissues, nappies, nappy wipes, etc.) |  |
| Attitudes (Non-Perishable Items) | If I were to buy more non-perishable products than I would use based on my usual frequency of shopping, it would be: | Item 1: 1 = “bad”, 7 = “good”  Item 2: 1 = “harmful”, 7 = “harmless”  Item 3: 1 = “unwise”, 7 = “wise” |
| Attitudes (Hygiene Products) | If I were to buy more hygiene products than I would use based on my usual frequency of shopping, it would be: | Item 1: 1 = “bad”, 7 = “good”  Item 2: 1 = “harmful”, 7 = “harmless”  Item 3: 1 = “unwise”, 7 = “wise” |
| Attitudes (Cleaning Products) | If I were to buy more cleaning products than I would use based on my usual frequency of shopping, it would be: | Item 1: 1 = “bad”, 7 = “good”  Item 2: 1 = “harmful”, 7 = “harmless”  Item 3: 1 = “unwise”, 7 = “wise” |
| Subjective Norms (Non-Perishable Items) | Regarding buying more non-perishable products than you would use based on your usual frequency of shopping during the COVID-19 pandemic, do you agree that...  Item 1: Most people who are important to me would approve of me buying more non-perishable products.  Item 2: Those people who are important to me think that I should buy more non-perishable products.  Item 3: Those people who are important to me would want me to buy more non-perishable products.  Item 4: Those people who are similar to me would buy more non-perishable products.  Item 5: Most people like me would buy more non-perishable products. | 1 = “strongly disagree”  2 = “disagree”  3 = “somewhat disagree”  4 = “neither agree nor disagree”  5 = “somewhat agree”  6 = “agree”  7 = “strongly agree” |
| Subjective Norms (Hygiene Products) | Regarding buying more hygiene products than you would use based on your usual frequency of shopping during the COVID-19 pandemic, do you agree that...  Item 1: Most people who are important to me would approve of me buying more hygiene products.  Item 2: Those people who are important to me think that I should buy more hygiene products.  Item 3: Those people who are important to me would want me to buy more hygiene products.  Item 4: Those people who are similar to me would buy more hygiene products.  Item 5: Most people like me would buy more hygiene products. | 1 = “strongly disagree”  2 = “disagree”  3 = “somewhat disagree”  4 = “neither agree nor disagree”  5 = “somewhat agree”  6 = “agree”  7 = “strongly agree” |
| Subjective Norms (Cleaning Products) | Regarding buying more cleaning products than you would use based on your usual frequency of shopping during the COVID-19 pandemic, do you agree that...  Item 1: Most people who are important to me would approve of me buying more cleaning products.  Item 2: Those people who are important to me think that I should buy more cleaning products.  Item 3: Those people who are important to me would want me to buy more cleaning products.  Item 4: Those people who are similar to me would buy more cleaning products.  Item 5: Most people like me would buy more cleaning products. | 1 = “strongly disagree”  2 = “disagree”  3 = “somewhat disagree”  4 = “neither agree nor disagree”  5 = “somewhat agree”  6 = “agree”  7 = “strongly agree” |
| Risk Perceptions (Non-Perishable Items) | Regarding buying more non-perishable products than you would use based on your usual frequency of shopping during the COVID-19 pandemic, do you agree that...  Item 1: It would be risky for me not to buy more non-perishable products  Item 2: If I do not buy more non-perishable products there would be risk involved | 1 = “strongly disagree”  2 = “disagree”  3 = “somewhat disagree”  4 = “neither agree nor disagree”  5 = “somewhat agree”  6 = “agree”  7 = “strongly agree” |
| Risk Perceptions (Hygiene Products) | Regarding buying more hygiene products than you would use based on your usual frequency of shopping during the COVID-19 pandemic, do you agree that...  Item 1: It would be risky for me not to buy more hygiene products  Item 2: If I do not buy more hygiene products there would be risk involved | 1 = “strongly disagree”  2 = “disagree”  3 = “somewhat disagree”  4 = “neither agree nor disagree”  5 = “somewhat agree”  6 = “agree”  7 = “strongly agree” |
| Risk Perceptions (Cleaning Products) | Regarding buying more cleaning products than you would use based on your usual frequency of shopping during the COVID-19 pandemic, do you agree that...  Item 1: It would be risky for me not to buy more cleaning products  Item 2: If I do not buy more cleaning products there would be risk involved | 1 = “strongly disagree”  2 = “disagree”  3 = “somewhat disagree”  4 = “neither agree nor disagree”  5 = “somewhat agree”  6 = “agree”  7 = “strongly agree” |
| Behavioural Automaticity (Non-Perishable Items) | Do you agree that buying more non-perishable products than you would use based on your usual frequency of shopping is something...  Item 1: I do automatically  Item 2: I do without having to consciously remember  Item 3: I do without thinking  Item 4: I start doing before I realise I am doing it | 1 = “strongly disagree”  2 = “disagree”  3 = “slightly disagree”  4 = “neither agree nor disagree”  5 = “slightly agree”  6 = “agree”  7 = “strongly agree” |
| Behavioural Automaticity (Hygiene Items) | Do you agree that buying more hygiene products than you would use based on your usual frequency of shopping is something...  Item 1: I do automatically  Item 2: I do without having to consciously remember  Item 3: I do without thinking  Item 4: I start doing before I realise I am doing it | 1 = “strongly disagree”  2 = “disagree”  3 = “slightly disagree”  4 = “neither agree nor disagree”  5 = “slightly agree”  6 = “agree”  7 = “strongly agree” |
| Behavioural Automaticity (Cleaning Items) | Do you agree that buying more cleaning products than you would use based on your usual frequency of shopping is something...  Item 1: I do automatically  Item 2: I do without having to consciously remember  Item 3: I do without thinking  Item 4: I start doing before I realise I am doing it | 1 = “strongly disagree”  2 = “disagree”  3 = “slightly disagree”  4 = “neither agree nor disagree”  5 = “slightly agree”  6 = “agree”  7 = “strongly agree” |
| Individual Difference Variables |  |  |
| COVID-19 Risk Perceptions | Do you agree that...  Item 1: There is a high chance of me getting COVID-19.  Item 2: I get sick more easily than other people my age.  Item 3: If I got COVID-19, there is a good chance that I would have trouble.  Item 4: If I got COVID-19, I would not be able to manage my daily activities | 1 = “strongly disagree”  2 = “disagree”  3 = “somewhat disagree”  4 = “neither agree nor disagree”  5 = “somewhat agree”  6 = “agree”  7 = “strongly agree” |
| Anxiety Sensitivity Index-3 (ASI-3; Taylor et al., 2007 | See Taylor et al. (2007) for item wording. | 0 = “very little”  1 = “a little”  2 = “Some”  3 = “Much”  4 = “very much” |
| Distress Tolerance Scale (DTS; Simons & Gaher, 2005) | See Simons and Gaher (2005) for item wording. | 1 = “strongly agree”  2 = “mildly agree”  3 = “agree and disagree equally”  4 = “mildy disagree”  5 = “strongly disagree” |
| Intolerance of Uncertainty (IUS-12; Charlton et al., 2007) | See Charlton et al. (2007) for item wording. | 1 = “not at all characteristic of me”  2  3 = “somewhat characteristic of me”  4  5 = “Entirely characteristic of me” |
| The Brief Self-control Scale (BSCS; Tangey et al., 2004) | See Tangey et al. (2004) for item wording. | 1 = “not at all”  2  3  4  5 = “Very much so” |
| The Hoarding Rating Scale-Interview (HRS; Tolin et al., 2010) | See Tolin et al. (2010) for item wording. | 0 = “Not difficult/no problem/none”  1  2 = “mild”  3  4 = “moderate”  5  6= “severe”  7  8 = “Extremely/extreme difficulty” |

Supplementary Appendix B

*Reliability Coefficients for Study Measures.*

| Variable | Reliability Coefficient |
| --- | --- |
| Attitude_NP | .91 |
| Attitude_H | .94 |
| Attitude_C | .92 |
| Subjective Norm_NP | .97 |
| Subjective Norm_H | .98 |
| Subjective Norm_C | .98 |
| Risk Perception_NP* | .93 |
| Risk Perception_H* | .95 |
| Risk Perception_C* | .95 |
| Behavioural Automaticity_NP | .95 |
| Behavioural Automaticity_H | .97 |
| Behavioural Automaticity_C | .97 |
| COVID Risk Perception | .81 |
| Intolerance of Uncertainty - P | .91 |
| Intolerance of Uncertainty - I | .90 |
| Distress Tolerance | .91 |
| Anxiety Sensitivity - Physical | .90 |
| Anxiety Sensitivity - Cognitive | .93 |
| Anxiety Sensitivity - Social | .89 |
| Self-control - Retraint | .78 |
| Self-control - Nonimpulsivity | .73 |
| Hoarding Rating | .87 |

* = Two-item scale reliabilities are Spearman rank order correlations.
All other reliability coefficients are Revelle’s ω.

Supplementary Appendix D

*Means and Standard Deviations for Study Variables.*

| Variable | M | SD |
| --- | --- | --- |
| NP_Beh | 2.53 | 1.62 |
| NP_Att | 3.61 | 1.77 |
| NP_Norms | 3.80 | 1.65 |
| NP_Risk | 3.76 | 1.80 |
| NP_Auto | 2.46 | 1.46 |
| H_Beh | 2.31 | 1.74 |
| H_Att | 3.57 | 1.83 |
| H_Norms | 3.76 | 1.69 |
| H_Auto | 2.33 | 1.47 |
| H_Risk | 3.67 | 1.81 |
| C_Beh | 2.06 | 1.68 |
| C_Att | 3.61 | 1.77 |
| C_Norms | 3.70 | 1.66 |
| C_Risk | 3.45 | 1.77 |
| C_Auto | 2.25 | 1.41 |
| DTS_Overall | 3.64 | .87 |
| ASI_Phys | 4.59 | 4.58 |
| ASI_Cog | 3.51 | 4.67 |
| ASI_Soc | 7.54 | 5.35 |
| IUS | 2.08 | .79 |
| SC_restraint | 2.80 | .90 |
| SC_nonimpuls | 3.60 | .85 |
| HRS | 6.34 | 6.41 |
| COVID_Risk | 3.52 | 1.37 |
| Age | 48.89 | 13.23 |
| People_in_household | 2.79 | 1.48 |
| Mins_to_shop_coded | 9.18 | 32.57 |

*Note:* Composite variables have been calculated to determine mean and standard deviation for variables included in the structural equation models.

Supplementary Appendix E

*Bivariate Correlations Between Study Variables*

|  | 1. | 2. | 3. | 4. | 5. | 6. | 7. | 8. | 9. | 10. | 11. | 12. | 13. | 14. | 15. | 16. | 17. | 18. | 19. | 20. | 21. | 22. | 23. | 24. | 25. | 26. | 27. | 28. | 29. | 30. |
| --- | --- | --- | --- | --- | --- | --- | --- | --- | --- | --- | --- | --- | --- | --- | --- | --- | --- | --- | --- | --- | --- | --- | --- | --- | --- | --- | --- | --- | --- | --- |
| 1. NP_Beh |  |  |  |  |  |  |  |  |  |  |  |  |  |  |  |  |  |  |  |  |  |  |  |  |  |  |  |  |  |  |
| 2. NP_Att | .58** |  |  |  |  |  |  |  |  |  |  |  |  |  |  |  |  |  |  |  |  |  |  |  |  |  |  |  |  |  |
| 3. NP_Norm | .56** | .64** |  |  |  |  |  |  |  |  |  |  |  |  |  |  |  |  |  |  |  |  |  |  |  |  |  |  |  |  |
| 4. NP_Risk | .61** | .61** | .69** |  |  |  |  |  |  |  |  |  |  |  |  |  |  |  |  |  |  |  |  |  |  |  |  |  |  |  |
| 5. NP_Auto | .31** | .38** | .39** | .38** |  |  |  |  |  |  |  |  |  |  |  |  |  |  |  |  |  |  |  |  |  |  |  |  |  |  |
| 6. H_Beh | .69** | .52** | .49** | .49** | .25** |  |  |  |  |  |  |  |  |  |  |  |  |  |  |  |  |  |  |  |  |  |  |  |  |  |
| 7. H_Att | .51** | .83** | .59** | .54** | .35** | .57** |  |  |  |  |  |  |  |  |  |  |  |  |  |  |  |  |  |  |  |  |  |  |  |  |
| 8. H_Norm | .49** | .57** | .85** | .60** | .34** | .53** | .64** |  |  |  |  |  |  |  |  |  |  |  |  |  |  |  |  |  |  |  |  |  |  |  |
| 9. H_Auto | .29** | .38** | .37** | .34** | .81** | .31** | .39** | .38** |  |  |  |  |  |  |  |  |  |  |  |  |  |  |  |  |  |  |  |  |  |  |
| 10. H_Risk | .53** | .56** | .64** | .80** | .34** | .58** | .59** | .68** | .39** |  |  |  |  |  |  |  |  |  |  |  |  |  |  |  |  |  |  |  |  |  |
| 11. C_Beh | .66** | .45** | .44** | .48** | .24** | .74** | .46** | .44** | .27** | .50** |  |  |  |  |  |  |  |  |  |  |  |  |  |  |  |  |  |  |  |  |
| 12. C_Att | .50** | .83** | .61** | .58** | .36** | .53** | .88** | .60** | .38** | .57** | .51** |  |  |  |  |  |  |  |  |  |  |  |  |  |  |  |  |  |  |  |
| 13. C_Norm | .50** | .57** | .86** | .63** | .35** | .48** | .58** | .86** | .38** | .63** | .48** | .64** |  |  |  |  |  |  |  |  |  |  |  |  |  |  |  |  |  |  |
| 14. C_Risk | .53** | .53** | .63** | .79** | .35** | .55** | .54** | .60** | .36** | .82** | .57** | .60** | .69** |  |  |  |  |  |  |  |  |  |  |  |  |  |  |  |  |  |
| 15. C_Auto | .28** | .36** | .35** | .35** | .84** | .28** | .37** | .34** | .89** | .36** | .28** | .38** | .39** | .41** |  |  |  |  |  |  |  |  |  |  |  |  |  |  |  |  |
| 16. DTS | -.09* | -.09* | -.08* | -.13** | -.14** | -.12** | -.08* | -.07* | -.20** | -.12** | -.13** | -.09* | -.05 | -.13** | -.17** |  |  |  |  |  |  |  |  |  |  |  |  |  |  |  |
| 17. ASI_P | .10** | .15** | .14** | .17** | .15** | .16** | .16** | .15** | .18** | .15** | .15** | .14** | .14** | .18** | .17** | -.49** |  |  |  |  |  |  |  |  |  |  |  |  |  |  |
| 18. ASI_C | .08* | .07 | .12** | .13** | .14** | .11** | .07* | .10** | .18** | .13** | .08* | .09** | .10** | .12** | .14** | -.55** | .63** |  |  |  |  |  |  |  |  |  |  |  |  |  |
| 19. ASI_S | .11** | .13** | .15** | .15** | .12** | .11** | .10** | .13** | .15** | .15** | .13** | .12** | .14** | .15** | .1** | -.53** | .61** | .62** |  |  |  |  |  |  |  |  |  |  |  |  |
| 20. IUS | .12** | .09* | .14** | .18** | .17** | .17** | .10** | .11** | .22** | .18** | .14** | .12** | .12** | .17** | .21** | -.67** | .53** | .64** | .66** |  |  |  |  |  |  |  |  |  |  |  |
| 21. Self-control_R | -.02 | -.07 | -.04 | -.07 | -.12** | -.02 | -.07 | -0.05 | -.16** | -.06 | .03 | -.06 | -.04 | -.06 | -.12** | .25** | -.25** | -.25** | -.27** | -.30** |  |  |  |  |  |  |  |  |  |  |
| 22. Self-control_N | .02 | -.01 | .01 | -.04 | -.10** | .05 | -.02 | .02 | -.13** | -.05 | .06 | -.00 | .03 | -.03 | -.08* | .22** | -.20** | -.23** | -.21** | -.24** | .50** |  |  |  |  |  |  |  |  |  |
| 23. HRS | .00 | .06 | .06 | .08* | .16** | .06 | .04 | .04 | .19** | .13** | .05 | .05 | 0.04 | .11** | .15** | -.29** | .33** | .29** | .30** | .37** | -.23** | -.26** |  |  |  |  |  |  |  |  |
| 24. COVID Risk | .20** | .16** | .21** | .23** | .10** | .17** | .16** | .21** | .12** | .23** | .19** | .16** | .21** | .24** | .13** | -.17** | .25** | .15** | .17** | .22** | -.15** | -.04 | .15** |  |  |  |  |  |  |  |
| 25. Age | -.04 | -.07 | -.13** | -.13** | -.05 | .02 | -.02 | -.09* | -.03 | -.09* | .02 | -.04 | -.11** | -.05 | .01 | .14** | .00 | -.14** | -.20** | -.26** | .16** | .13** | .01 | -.02 |  |  |  |  |  |  |
| 26. Gender | .05 | .03 | .05 | -.01 | -.05 | .05 | .04 | .04 | -.03 | -.01 | .01 | .02 | .02 | -.04 | -.04 | -.09* | .07* | .04 | .03 | .04 | -.02 | .17** | -.04 | .07* | -.10** |  |  |  |  |  |
| 27. Household Size | .01 | .07 | .08* | .08* | .02 | .04 | .10** | .09* | -.00 | .10** | .05 | .12** | .12** | .12** | .03 | .03 | -.06 | .01 | .00 | .01 | .03 | .13** | -.05 | -.04 | -.28** | .12** |  |  |  |  |
| 28. Education | .07* | .06 | .07* | .08* | -.05 | .03 | .01 | .07 | -.04 | .08* | .07 | .04 | .11** | .07 | -.07* | .09** | -.11** | -.08* | -.04 | -.05 | .07* | -.02 | -.03 | -.05 | -.16** | .01 | .01 |  |  |  |
| 29. Minutes to Supermarket | .06 | .02 | .00 | .00 | .01 | -.01 | .03 | .00 | .02 | .01 | -.01 | .03 | .00 | .01 | .02 | -.00 | -.02 | -.02 | -.04 | -.02 | -.01 | .02 | .01 | .03 | .08* | .03 | -.05 | -.03 |  |  |
| 30. Income | .02 | .00 | .00 | .04 | .01 | .00 | .00 | .01 | -.02 | .06 | -.02 | .01 | .01 | .04 | .02 | .15** | -.14** | -.12** | -.05 | -.14** | .00 | .01 | -.13** | -.09* | -.19** | .00 | .20** | .17** | -.07* |  |

Note: * = *p* < .05; ** = *p* < .01

Supplementary Appendix F

*Factor Loadings*

|  | Model 1 – Non-Perishable | Model 2 – Hygiene | Model 3 – Cleaning |
| --- | --- | --- | --- |
| Construct | Factor Loading | Factor Loading | Factor Loading |
| Attitude  Item 1  Item 2  Item 3 | .93  .80  .89 | .96  .86  .90 | .94  .83  .91 |
| Subjective Norms  Item 1  Item 2  Item 3  Item 4  Item 5 | .90  .86  .86  .83  .81 | .94  .92  .93  .82  .80 | .93  .93  .92  .82  .80 |
| Risk Perceptions  Item 1  Item 2 | .91  .95 | .94  .97 | .94  .96 |
| Automaticity  Item 1  Item 2  Item 3  Item 4 | .88  .96  .87  .71 | .91  .97  .88  .79 | .90  .96  .90  .93 |
| COVID Risk Perception  Item 1  Item 2  Item 3  Item 4 | .45  .64  .86  .68 | .45  .64  .86  .68 | .45  .64  .87  .68 |
| Intolerance of Uncertainty - P  Item 7  Item 8  Item 10  Item 11  Item 18  Item 19  Item 21 | .76  .66  .64  .70  .73  .74  .70 | .76  .66  .64  .70  .73  .74  .70 | .76  .66  .64  .70  .73  .74  .70 |
| Intolerance of Uncertainty – I  Item 9  Item 12  Item 15  Item 20  Item 25 | .82  .73  .83  .76  .75 | .82  .73  .83  .76  .76 | .82  .73  .83  .76  .75 |
| Distress Tolerance  Subscale 1  Subscale 2  Subscale 3  Subscale 4 | .83  .90  .84  .69 | .83  .90  .84  .69 | .83  .90  .74  .69 |
| Anxiety Sensitivity – Physical  Item 3  Item 4  Item 7  Item 8  Item 12  Item 15 | .72  .65  .76  .80  .78  .59 | .72  .65  .76  .80  .78  .59 | .72  .65  .76  .80  .78  .59 |
| Anxiety Sensitivity – Cognitive  Item 2  Item 5  Item 10  Item 14  Item 16  Item 18 | .70  .67  .83  .85  .83  .80 | .70  .67  .83  .85  .83  .80 | .70  .67  .83  .85  .83  .80 |
| Anxiety Sensitivity – Social  Item 1  Item 6  Item 9  Item 11  Item 13  Item 17 | .51  .79  .85  .61  .77  .51 | .51  .79  .85  .61  .77  .51 | .51  .79  .85  .61  .77  .51 |
| Self-control – Restraint  Item 1  Item 2R  Item 7R  Item 8 | .64  .66  .74  .50 | .64  .66  .74  .50 | .64  .66  .75  .50 |
| Self-control – Nonimpulsivity  Item 5R  Item 9R  Item 12R  Item 13R | .55  .53  .70  .60 | .55  .53  .70  .60 | .55  .52  .70  .60 |
| Hoarding Rating  Item 1  Item 2  Item 3  Item 4  Item 5 | .75  .66  .62  .74  .76 | .82  .73  .83  .76  .76 | .75  .67  .62  .74  .76 |

*Note*: Standardised factor loadings
