# Supplementary material for: Is it time to stock up? Understanding panic buying during the COVID-19 pandemic
Source: Aust J Psychol. 2023 Mar 16;75(1):2180299. doi: 10.1080/00049530.2023.2180299 (PMC12175686; doi:10.1080/00049530.2023.2180299)
Supplement: Appendix G [file RAUP_A_2180299_SM9233.pdf]

Supplementary Appendix G

Model Diagrams Depicting Structure, Factor Loadings, Covariances, and Standardised Path Coefficients

Model 1 Predicting Non-Perishable Food Items

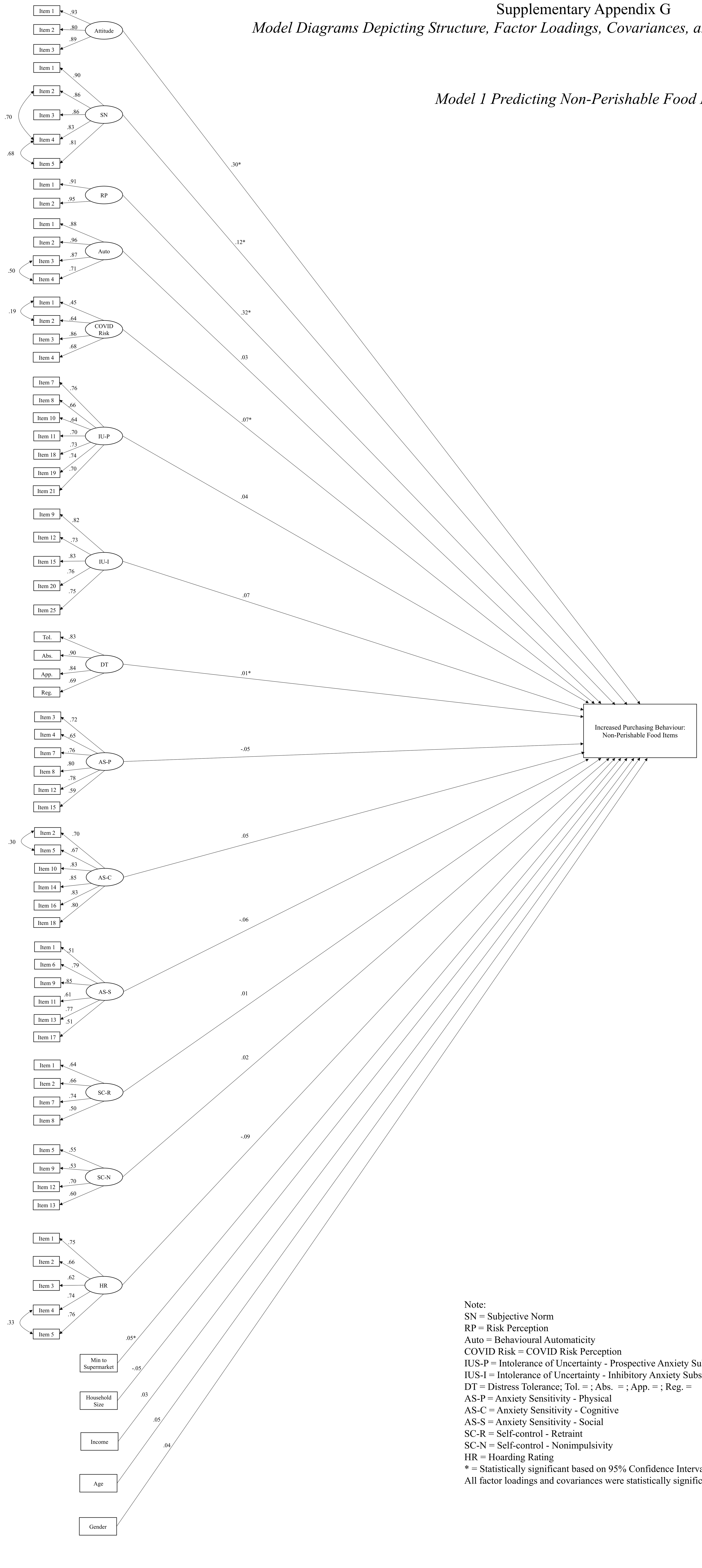

Note:
SN = Subjective Norm
RP = Risk Perception
Auto = Behavioural Automaticity
COVID Risk = COVID Risk Perception
IUS-P = Intolerance of Uncertainty - Prospective Anxiety Subscale
IUS-I = Intolerance of Uncertainty - Inhibitory Anxiety Subscale
DT = Distress Tolerance; Tol. = ; Abs. = ; App. = ; Reg. =
AS-P = Anxiety Sensitivity - Physical
AS-C = Anxiety Sensitivity - Cognitive
AS-S = Anxiety Sensitivity - Social
SC-R = Self-control - Retraint
SC-N = Self-control - Nonimpulsivity
HR = Hoarding Rating
* = Statistically significant based on 95% Confidence Intervals not encompassing zero.
All factor loadings and covariances were statistically significant at $p < .001$

Model 2 Predicting Hygiene Products

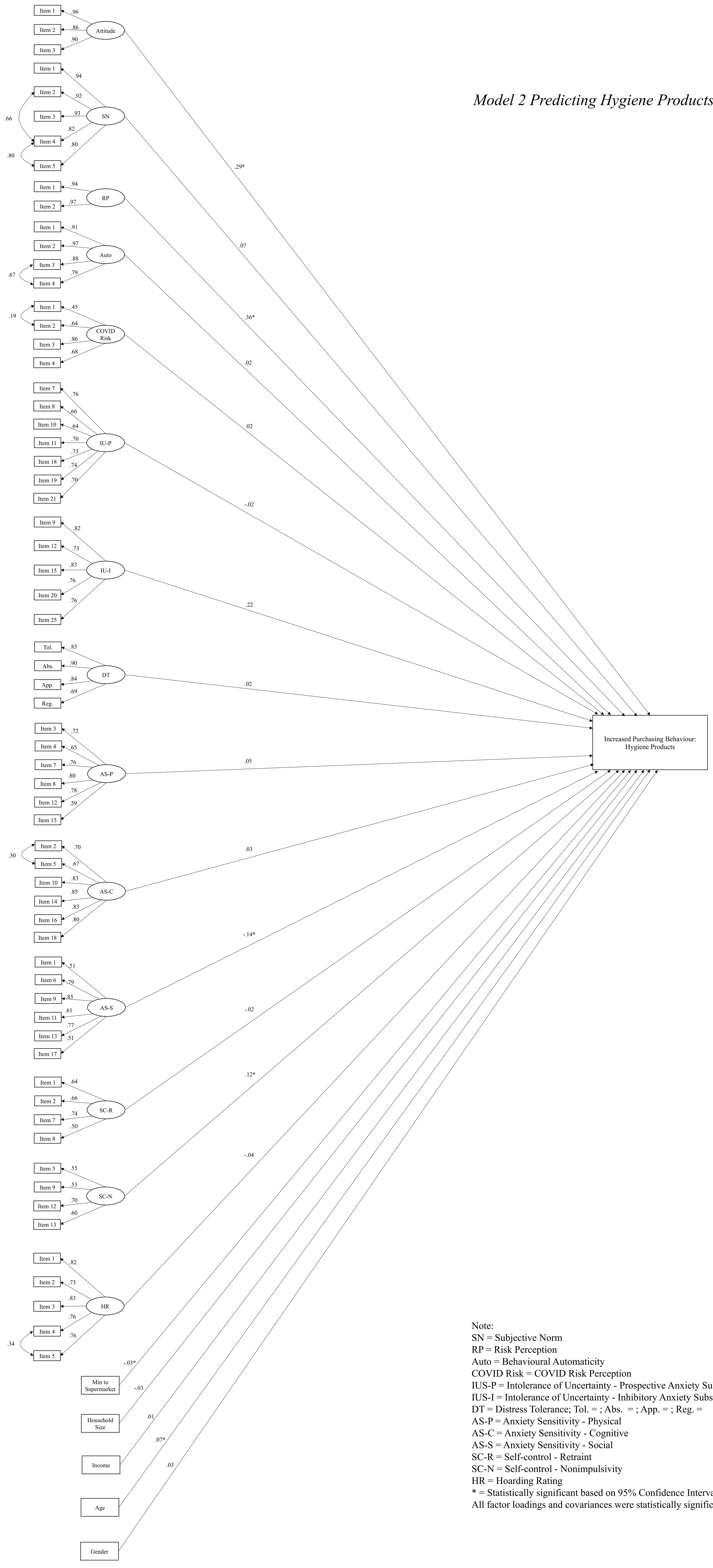

Note:
SN = Subjective Norm
RP = Risk Perception
Auto = Behavioural Automaticity
COVID Risk = COVID Risk Perception
IUS-P = Intolerance of Uncertainty - Prospective Anxiety Subscale
IUS-I = Intolerance of Uncertainty - Inhibitory Anxiety Subscale
DT = Distress Tolerance; Tol. = ; Abs. = ; App. = ; Reg. =
AS-P = Anxiety Sensitivity - Physical
AS-C = Anxiety Sensitivity - Cognitive
AS-S = Anxiety Sensitivity - Social
SC-R = Self-control - Retraint
SC-N = Self-control - Nonimpulsivity
HR = Hoarding Rating
* = Statistically significant based on 95% Confidence Intervals not encompassing zero.
All factor loadings and covariances were statistically significant at $p < .001$

Model 3 Predicting Cleaning Products

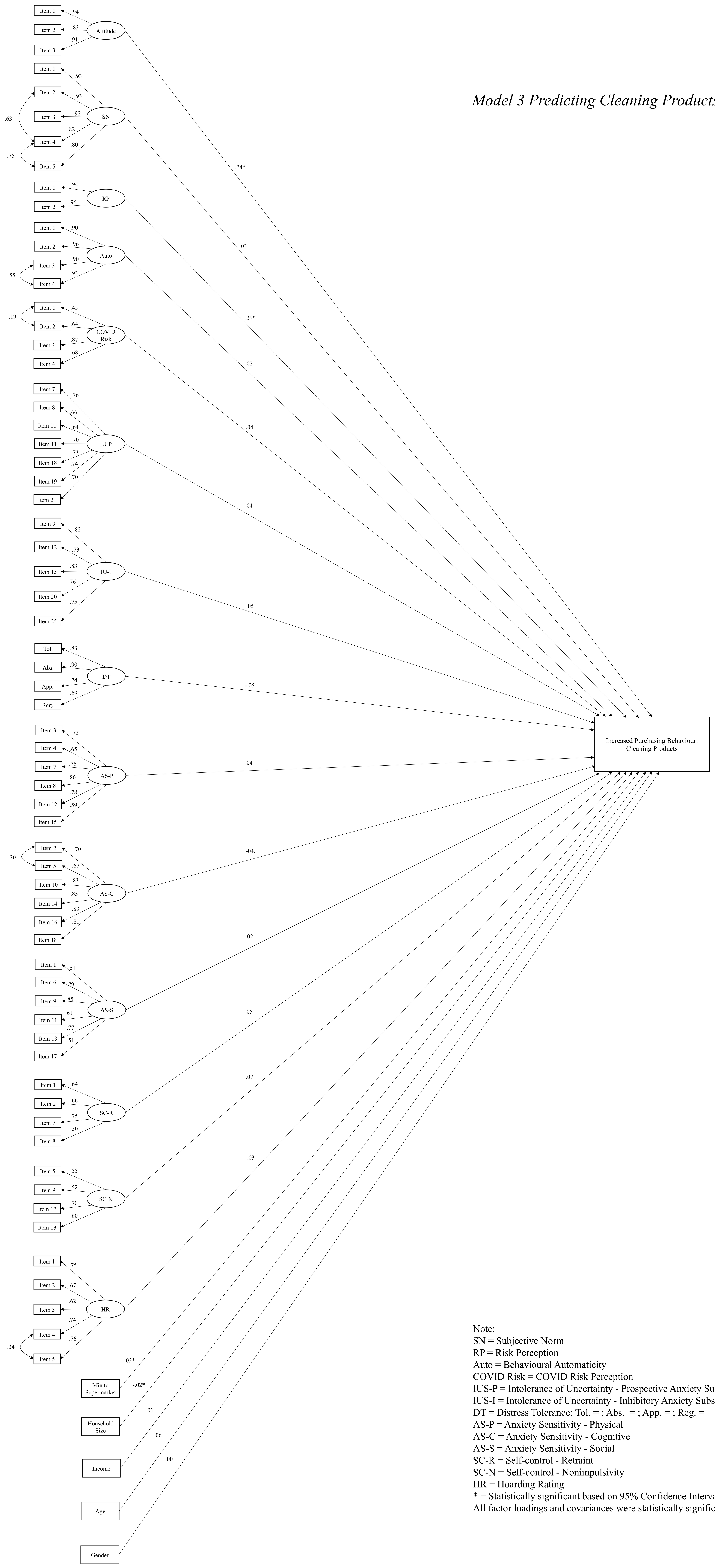

Note:
SN = Subjective Norm
RP = Risk Perception
Auto = Behavioural Automaticity
COVID Risk = COVID Risk Perception
IUS-P = Intolerance of Uncertainty - Prospective Anxiety Subscale
IUS-I = Intolerance of Uncertainty - Inhibitory Anxiety Subscale
DT = Distress Tolerance; Tol. = ; Abs. = ; App. = ; Reg. =
AS-P = Anxiety Sensitivity - Physical
AS-C = Anxiety Sensitivity - Cognitive
AS-S = Anxiety Sensitivity - Social
SC-R = Self-control - Retraint
SC-N = Self-control - Nonimpulsivity
HR = Hoarding Rating
* = Statistically significant based on 95% Confidence Intervals not encompassing zero.
All factor loadings and covariances were statistically significant at $p < .001$
